# The Student's Surgery

**Published:** 1890-03

**Authors:** 


*The Student's Surgery.* By FREDERICK JAMES GANT, F.R.C.S. Pp. 817. London: BAILLIÈRE & Co. 1890.

"Still another Student's Surgery!" we grumble as we open this rather striking little work; and we proceed to grumble generally about the superficiality of the age we live in, the tendency to skim the surface instead of digging the depths, and we make specific applications. Surgery grows and grows; and surgical "students'" books get smaller and smaller. Instead of trying to educate our men up to the requirements of their art, we bring our art down to their capacities. We want to recruit students from among university graduates, and we give them pabulum suited for boys leaving dames' schools! That is our grumbling, however.

This book is not so bad as that. Small print, broad pages, and thin paper make up a volume that contains a good deal, if it is not bulky. And of the matter: well, we may honestly say that the compounding of it must have represented an immense amount of labour—which labour, we fear we must add, would have been, to the detriment neither of the matter nor the author, far better divided. There is no man alive—and there never will live another man—who, alone and unaided, will present the best possible compendium of surgery. It must be divided up. On several subjects with which we are specially familiar, it would be easy to point out shortcomings; but we feel it would be ungracious to do so. As a total individual effort the work represents a wide knowledge, great industry, and much judgment; but as a text-book for students we cannot recommend it.

The get-up of the book is good—with the exception of the illustrations. Granting that the illustrations may be mutilated reproductions of antiquated engravings, admitting that the artist engaged for a medical work may be ignorant of most of the elementary principles of art, we still feel we must rebel against the necessity of a man who has a fracture of the neck of the femur having also a squint (p. 303). And on the opposite page there is a drawing of a femur (signed "Author") which is worthy of study, for it is an extraordinary bone. In Fig. 143, the size of an organ along which a man is passing a sound is, by measurement, equal to that of the wrist of the surgeon who is officiating. All through, the illustrations are as bad as we have ever seen in a surgical work—and that is saying a good deal!

